# Clinical Efficacy of Two Different Low-Level Laser Therapies for the Treatment of Trigeminal Neuralgia: A Randomized, Placebo-Controlled Trial

**DOI:** 10.3390/jcm13226890

**Published:** 2024-11-15

**Authors:** İrem Karagözoğlu, Nermin Demirkol, Özge Parlar Öz, Gökçe Keçeci, Beste Çetin, Mutlu Özcan

**Affiliations:** 1 Faculty of Dentistry, Department of Prosthodontics, Gaziantep University, Gaziantep 27310, Turkey; nhamdemirci@gantep.edu.tr (N.D.); ozgeparlar@gantep.edu.tr (Ö.P.Ö.); 2 Faculty of Dentistry, Department of Prosthodontics, Kahramanmaraş Sütçü İmam University, Kahramanmaraş 46050, Turkey; gokcekececi@ksu.edu.tr (G.K.); bestecetin@ksu.edu.tr (B.Ç.); 3 Clinic of Masticatory Disorders and Dental Biomaterials, Center for Dental Medicine, University of Zurich, 8032 Zurich, Switzerland; mutlu.ozcan@zzm.uzh.ch

**Keywords:** clinical trial, trigeminal neuralgia, low-level laser therapy, diode laser, neodymium-doped yttrium aluminum garnet lasers

## Abstract

**Background:** Trigeminal neuralgia (TN) is a disease that causes severe pain that can seriously affect the quality of life. This study aimed to compare the effectiveness of two different low-level laser therapies (LLLT) as alternatives to medical treatment to reduce pain and improve the quality of life in patients with TN. **Methods:** A total of 45 patients were randomly divided into 3 groups. In the first group, a new-generation diode laser (GRR laser) was applied at predetermined points in the trigeminal nerve line. In the second group, a low-level neodymium-doped yttrium aluminum garnet (Nd:YAG) laser was applied along the affected nerve line. The placebo group received the same protocol with a Nd:YAG laser without the device switched on. The scores were recorded pre- and post-treatment using the Brief Pain Inventory-Facial (BPI-facial) scale. **Results:** A statistically significant difference was found between the pre- and post-treatment values of all four variables in the GRR laser, Nd:YAG laser, and placebo groups. When the post-treatment values were compared, statistically significant differences were found between the groups in pain frequency, pain intensity, and interference in facial-specific activities, but no differences were found in general activities. **Conclusions:** Both LLLTs can be considered alternative treatment modalities for TN, but the GRR laser treatment was more effective than the Nd:YAG laser treatment in reducing pain and improving the quality of life in patients with TN.

## 1. Introduction

Trigeminal neuralgia (TN), classified as neuropathic facial pain, is defined as sudden, transient but highly intense and usually unilateral facial pain along the trigeminal nerve [1]. Patients with severe facial pain usually visit the dentist first because this pain is often confused with a toothache. The most common cause of TN is the compression of the trigeminal nerve by a nearby blood vessel, often the superior cerebellar artery (Classic TN) [2]. Less common causes include tumors, multiple sclerosis, and other neurological conditions (Secondary TN) [2]. TN is diagnosed using a detailed medical history, imaging (Magnetic Resonance Imaging—MRI), and neurological examination after dental factors have been eliminated. If an MRI does not identify a tumor that compresses the nerve, the condition is defined as TN of unknown cause (Idiopathic TN) [3]. The International Headache Society has defined several diagnostic criteria for idiopathic TN [4]. These are described as pain that lasts from a few seconds to 2 min and is distributed over one or more branches of the trigeminal nerve; the presence of pain or trigger points during daily activities such as talking, eating, washing one’s face, or brushing one’s teeth; and pain like an electric shock, stabbing, stinging, or sharp pain.

The aim of TN treatment is to reduce or eliminate the frequency and intensity of this pain in the jaw and facial areas. This pain significantly reduces the patients’ quality of life. The first step in TN treatment is the use of medication [2,3]. The effect of carbamazepine, an anticonvulsant drug, has been proven to relieve neuropathic pain, but this drug has many side effects, especially a decrease in white blood cells and serum sodium levels [5]. In addition, drug therapy cannot completely cure TN, and drugs have a symptomatic effect on pain relief during the period of use. Medication is started at a low dose, and the dose may be increased to 1200 mg until the pain disappears [3]. In cases where medical treatment is not effective in controlling pain, interventional treatments such as peripheral nerve intervention, botulinum toxin, gamma knife radiosurgery, and microvascular decompression are applied, but the risk of complications with interventional treatments is high [2,3]. Peripheral alcohol or glycerol injection was one of the most common surgical methods [3]. These methods significantly reduce the pain levels, but they require technical precision and have some complications. The complications include regional swelling, loss of sensation, and paresthesia [3]. As a result, new treatments are being investigated to reduce pain, which can severely affect the patients’ quality of life.

In recent years, low-level laser therapy (LLLT), or, also, photobiomodulation therapy, have been widely used in dentistry and medicine for pain relief. Compared with high-intensity laser treatments, this treatment method is used to promote biological changes without causing temperature changes in the tissue [6,7]. With LLLT, low-level light penetrates tissue and reaches neural tissue, stimulating ‘neurogenesis’ and the production of nervous system cells from neural stem cells [8]. Another effect of LLLT is the neuroprotection of nerve tissue [9]. The low level of light absorbed by the tissue increases adenosine triphosphate (ATP) production in the mitochondria. The increased energy in intracellular organelles can repair some neuronal damage or prevent further damage [10]. In clinical studies, it has been reported that LLLT improves myelin synthesis capacity and increases nerve function in damaged nerves [11]. Laser energy is known to increase the repolarization of the cell membrane, reduce the release of substances that stimulate pain receptors, and significantly increase the pain threshold by stimulating endorphin synthesis [12]. Therefore, it can be effective for treating peripheral pain in the facial area, such as TN. The LLLT is a noninvasive method for controlling pain in patients with TN. It is not possible to completely repair the degeneration of nerve axons, but it has been reported that patients treated with low-level lasers have been pain-free for 2–3 years [2,13]. Considering the side effects of the drugs used to treat TN and the serious complications of invasive methods, LLLT with no reported side effects may be a therapeutic modality or useful in combination with existing treatments. The LLLT can help to reduce the pain, so the dose and side effects of the medication used can be reduced and may not require invasive surgical procedures. However, there is no clear consensus on the type, wavelength and duration of low-level lasers that should be used.

The most common symptom of TN is pain, and the aim of treatment is to reduce or eliminate pain. The accurate measurement of chronic pain is critical for determining the effectiveness of surgical and medical treatments. To measure this type of pain, carefully designed instruments with proven reliability and validity are needed. In general, pain severity has been assessed in trials using the visual analog scale (VAS) or categorical numerical rating scale (NRS) [14,15,16,17]. Both scales measure a single aspect of pain, namely, the intensity of the pain at the time that the patient completed the questionnaire. However, it is also necessary to assess the frequency of pain, the daily disability caused by the pain, and its psychosocial impact. For this purpose, the Brief Pain Inventory-Facial (BPI-facial) has been used in recent studies to measure pain in patients with TN and has been reported to be a reliable method [18]. Pain intensity (PI), interference in general activities (IGA), and interference in face-specific activities (IFA) are evaluated with the BPI-facial scale. Thus, both types of pain are evaluated multidimensionally, and improvements in the patients’ quality of life can be evaluated.

Guidelines for the diagnosis and management of patients with TN advocate a multidisciplinary team approach to improve the care of patients with acute and chronic TN [4]. In addition to the medical treatment usually prescribed by neurologists, LLLT is a good alternative to achieve more rapid pain relief or to reduce the dose of medication used. The present study aimed to compare the effectiveness of two different LLLTs in patients diagnosed with TN and started on medical treatment. The null hypothesis of the study was that low-level laser treatments would not result in clinically significant improvements compared to those in the control group.

## 2. Materials and Methods

This randomized controlled clinical trial was approved by the Ethics Committee of Gaziantep University (Decision number: 2022/419, Date for approval: 4 January 2023) and was performed in accordance with the principles of the Declaration of Helsinki. A power analysis was performed with G*power version 3.1.9.2. Using an effect size of 0.45, 80% power, and a two-sided 5% significance level, the minimum sample size was calculated to be 45 according to a previous study [14]. It was determined that 45 participants would be included in the study (*n* = 15). Each participant signed an informed consent form before the commencement of the treatment. Information regarding the clinical trial registration is available at www.ClinicalTrials.gov, accessed on 27 May 2024 (identifier NCT06440356). The patients were selected from those attending the Dentistry Faculty of Gaziantep University. The patients who were diagnosed with TN by neurologists and commenced medical treatment were included in the study. The inclusion and exclusion criteria were as follows.

Inclusion criteria:

- Patients diagnosed with idiopathic TN as defined by the International Headache Society [4];
- Patients diagnosed with idiopathic TN and receiving medical treatment (carbamazepine, etc.);
- Patients with unilateral, severe, sudden onset of facial pain along the branches of the trigeminal nerve;
- Patients who had not previously received any interventional treatment for TN;
- Patients who were recently diagnosed and started on a first dose of carbamazepine and its derivatives.

Exclusion criteria:

- Patients who were diagnosed with type 2 (atypical, symptomatic) TN as defined by the International Headache Society [4];
- Patients with etiologies such as tumors, multiple sclerosis, or neurovascular compression on radiography;
- Pregnant women;
- Patients with systemic diseases such as diabetes, cardiovascular disease, hypertension, etc.;
- Patients who had been previously diagnosed and treated with any type of TN therapy.

### 2.1. Randomization Method

**Figure 1:**
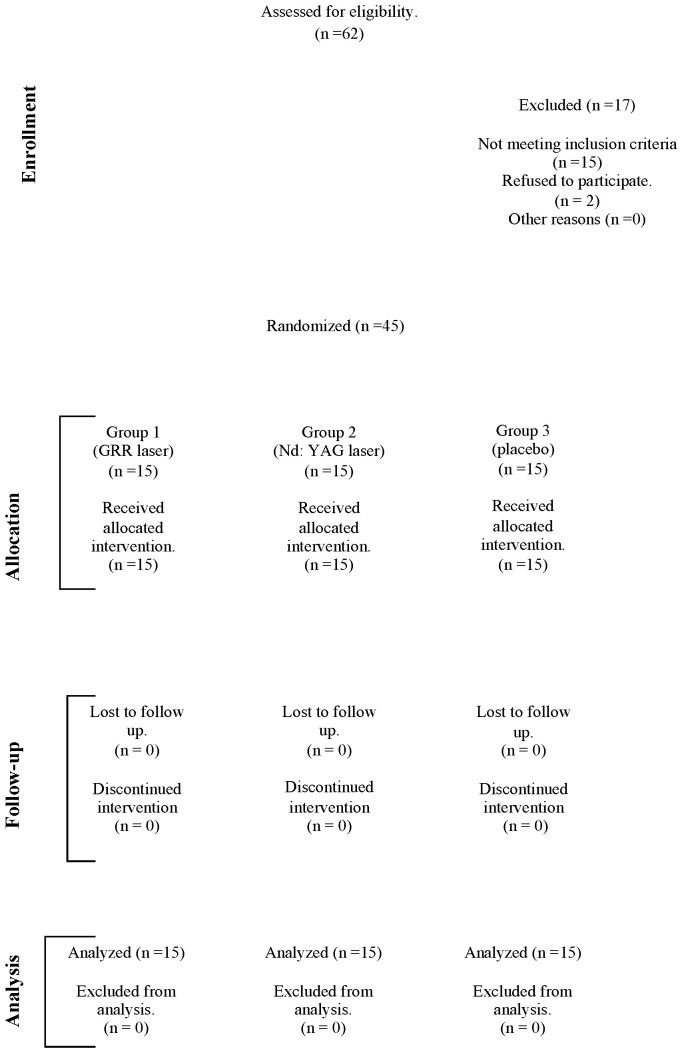
Consort flow diagram.

A total of 62 patients were examined, and 45 patients fulfilling the inclusion criteria were divided into 3 groups (15 patients in each group). The free Research Randomizer website, developed for researchers, was used for the randomization procedure. The randomized patients were designed as parallel group trial. The study was carried out in accordance with the CONSORT (Consolidated Standards of Reporting Trials) guidelines. A plot of patient enrollment and randomization throughout the trial is shown in Figure 1 (CONSORT flow diagram). According to the research randomization results, the numbers in the first set of 15 are defined as Group 1 (GRR laser group), the numbers in the second set of 15 are defined as Group 2 (Nd:YAG laser group), and the numbers in the third set of 15 are defined as Group 3 (placebo group).

### 2.2. Treatment Protocol

In the first group, a GRR laser (GRR laser 2000, Ankara, Turkey) was applied to the patients. The GRR laser is a combination of a 22 mW gallium–aluminum–arsenide (GaAlAs) infrared laser with a wavelength of 904 nm and a 10 mW red laser with a wavelength of 650 nm. The GRR laser has a cluster unit with numerous diodes and a rectangular probe which can be applied regionally. The rectangular probe, with an energy transfer of 16 J during a 1 min application, has an external surface area of 60 mm and an internal surface area of 30 mm.

**Figure 2:**
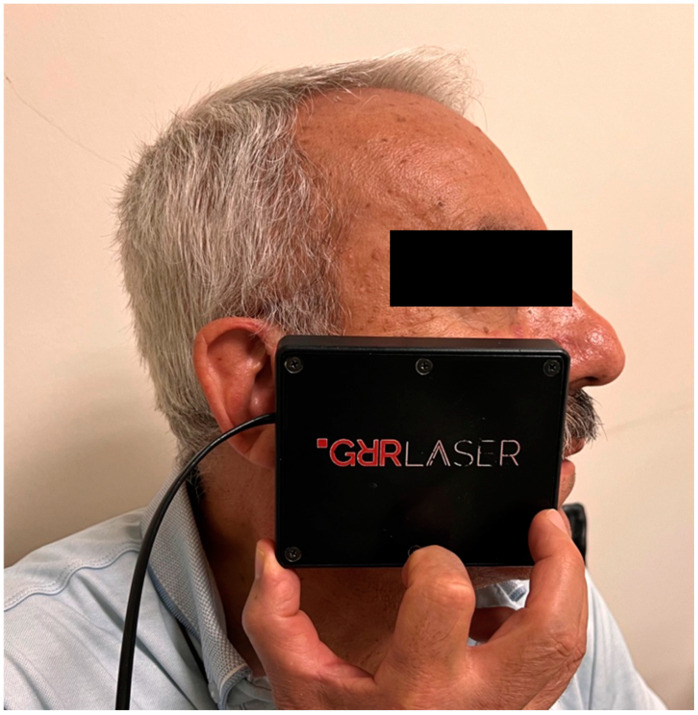
Application of GRR laser.

The manufacturer recommends a two-stage approach, consisting of 25 sessions, for pain, inflammation, and healing in any area. In this study, the first 15 sessions of treatment were performed according to the manufacturer’s instructions: the first 5 sessions were performed once per day, and the remaining 10 sessions were performed once every other day (1st stage). The treatment was suspended for 1 week to allow the body to recover. Among the remaining 10 sessions, the first 5 sessions were performed once per day, and 5 sessions were performed every other day (2nd stage). For each application, the rectangular probe of the device was used to irradiate the area where the trigeminal nerve was affected (ophthalmic, maxillary, or mandibular nerve) for 5 min (Figure 2).

**Figure 3:**
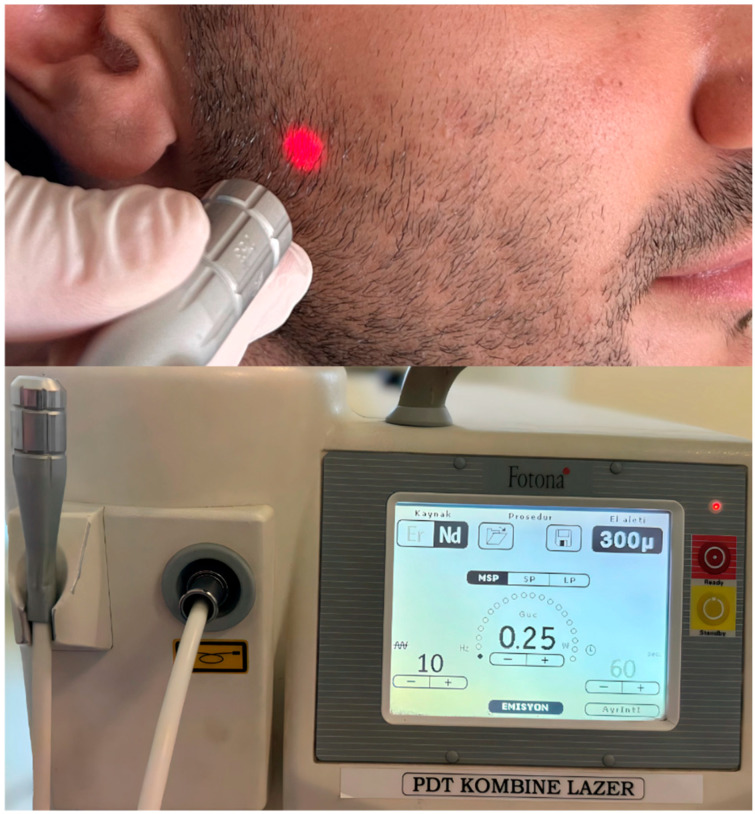
Application of Nd:YAG laser and low-level laser parameters.

In the second group, a neodymium-doped yttrium aluminum garnet laser (Nd:YAG laser, Fidelis Plus 3, Fotona, Slovenia) with a single wavelength of 1064 nm was applied. After the laser unit was switched on, the LLLT preset was selected. The output power was set to 0.25 W, the duration to 20 s, and the energy density to 8 J/cm^2^. The tip of the 0.9 mm diameter LLLT probe was positioned 1 cm along the painful nerve line in noncontact pulse mode. A total of 12 sessions, 3 sessions per week, were applied, according to reference studies [11,14,16]. According to the affected nerve region (ophthalmic, maxillary, or mandibular nerve) of each patient, Nd:YAG laser was applied to 3 points along the nerve line for a total of 60 s, with each point being used for 20 s (Figure 3).

The patients in the third group received emission-free laser treatment. With the same procedure with the Nd:YAG laser, a placebo treatment was performed with the device on, such that laser beams were visible but not active. The patients in all three groups were informed not to take any analgesics during treatment.

### 2.3. Assessment Protocol

For the patient assessment procedure, a data collection form was prepared according to the BPI-facial form [18]. At the first treatment session, after the patients’ demographic data were recorded, the pain frequency (number of pain attacks in the previous week) and BPI-facial data were recorded. The BPI-facial consists of three parts. In the first part, pain intensity was assessed. The patients were asked about their current pain, worst pain over the previous week, least pain over the previous week, and average pain over the previous week. The patients were asked to answer the questions on the NRS (0: no pain, 10: the worst pain you can imagine). In the second part, the IGA due to pain was assessed with 7 questions (0: no inhibition, 10: complete inhibition). In the third part, the IFA was assessed with 7 questions, and the total score was recorded (0: no inhibition, 10: complete inhibition). The same assessment procedure was repeated at the end of treatment.

### 2.4. Statistical Analysis

The program SPSS version 29.0 was used for the analysis of continuous data. The Kolmogorov—Smirnov and Shapiro—Wilk tests were used to test whether there was a difference between the distributions of the data and the normal distribution. The difference between the means of the dependent continuous variables was analyzed via the paired samples *t* test. The one-way ANOVA was used to compare variables with a normal distribution between more than two independent groups. Finally, the relationships between two categorical variables were analyzed via the chi-square test. *p* < 0.05 was considered statistically significant.

## 3. Results

A total of 45 patients with a mean age of 46.09 years (26 females, 19 males) participated in this study. When the mean age was compared between the groups, no statistically significant difference was found. When the pre- and post-treatment values were compared in the GRR laser group, a statistically significant difference was found between the pre- and post-treatment values of all four variables (Table 1).

In the Nd:YAG laser group, a statistically significant difference was found between the pre- and post-treatment values for all four variables (Table 2). Additionally, in the placebo group, there was a statistically significant difference between the PF, PI, IGA, and IFA pre- and post-treatment values (Table 3).

**Figure 4:**
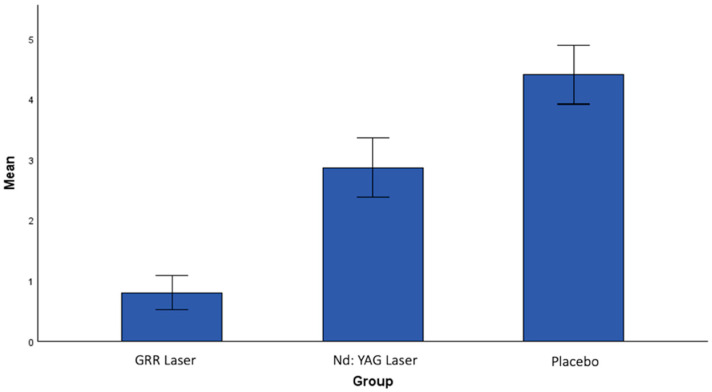
The mean values of pain frequency after treatment in all groups.

When the post-treatment values were compared between the groups, a statistically significant difference was found. When the post-treatment pain frequency (PF2) scores were analyzed, a statistically significant difference was found between the GRR laser group and the Nd:YAG laser group (*p* = 0.004). A statistically significant difference was also found between the GRR laser group and the placebo group (*p* < 0.001). The reduction in pain frequency was greatest in the GRR laser group, followed by the Nd:YAG laser group, and lowest in the placebo group (Figure 4).

**Figure 5:**
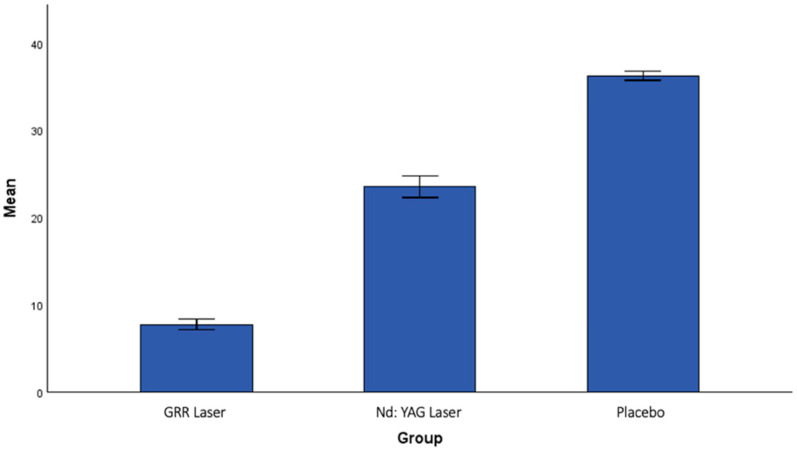
The mean values of pain intensity after treatment in all groups.

When the post-treatment pain intensity (PI2) values were analyzed, a statistically significant difference was found between the GRR laser group and the Nd:YAG laser group (*p* < 0.001) and between the GRR laser group and the placebo group (*p* < 0.001). The reduction in pain intensity was greatest in the GRR laser group, followed by the Nd:YAG laser group, and lowest in the placebo group (Figure 5).

**Figure 6:**
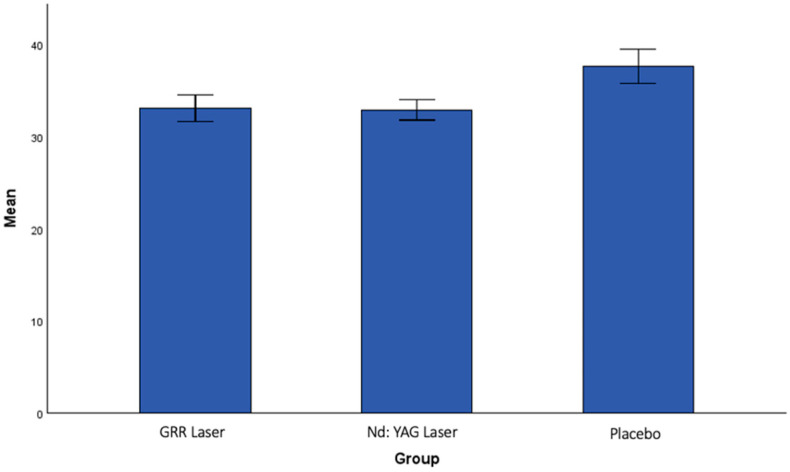
The mean values of interference in IGA after treatment in all groups.

**Figure 7:**
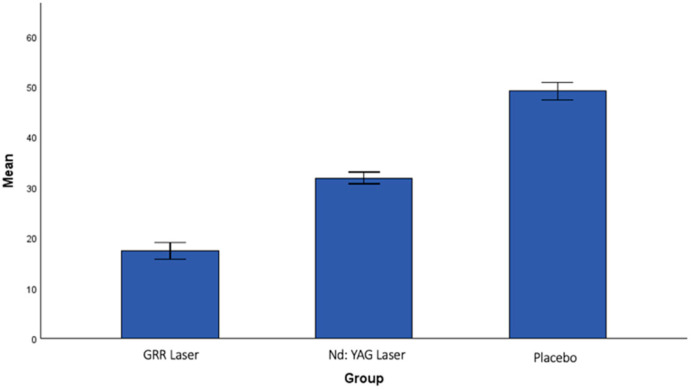
The mean values of interference in IFA after treatment in all groups.

There was no statistically significant difference between the groups in the analysis of the post-treatment interference in general activities (IGA2) (Figure 6). When the post-treatment interference in face-specific activities (IFA2) values were analyzed, a statistically significant difference was found between the GRR laser group and the Nd:YAG laser group (*p* < 0.001). A statistically significant difference was also found between the GRR laser group and the placebo group (*p* < 0.001). The greatest reduction in IFA scores was observed in the GRR laser group, followed by the Nd:YAG laser group, with the lowest reduction observed in the placebo group (Figure 7).

## 4. Discussion

TN is a disorder that causes very severe pain with a significant impact on the quality of life. Although there are different treatment options, the first step in treatment is the use of medication [2]. In addition to drug treatment, several palliative treatments exist. The LLLT is also used in addition to medical treatment to reduce symptoms and improve quality of life. The number of studies evaluating the effect of LLLT on TN is limited. Several studies have evaluated its efficacy in idiopathic facial pain or myofascial pain [16,19,20]. While some of these trials reported a positive effect of LLLT, others reported no significant difference between the treatment and control groups [16,20]. A clinical trial evaluating the efficacy of LLLT in patients with TN reported a significant reduction in pain intensity compared with the control group [14]. Like our study, this study included LLLT in addition to medical treatment and a control group. In our study, both LLLTs caused a decrease in all the parameters compared with control group. Therefore, the null hypothesis of the study is rejected. In their study, Rezazadeh et al. reported that the short-term application of LLLT was effective in relieving pain in patients with TN [17].

Laser types, wavelengths, and application times vary in studies of pain relief. There is no definitive parameter for this subject. In their study, Al-Azab et al. [15] evaluated the effects of LLLT and electromagnetic therapy (EMG) treatments on TN. In this study, a low-power helium–neon laser with a wavelength of 830 nm and a beam intensity of 150–170 mW/cm^2^ was used as a low-level laser, and it was reported that LLLT was more effective than EMG in reducing pain. In our study, two different types of lasers and laser parameters were used, and both were effective in reducing pain. Helium–neon lasers (632.8 nm), diode lasers (830 nm, 890 nm, 670 nm), GaAs lasers (980 nm), and GaAlAs diode lasers (810 nm) are generally used for LLLT in TN [16,21,22]. In our study, we applied a GaAlAs diode laser with two wavelengths (650 nm and 904 nm) and a Nd:YAG laser (1064 nm). The efficacy of the GaAlAs laser in postherpetic neuralgia has been investigated in various studies, and it was found to be effective in reducing pain [23,24]. However, in another study, no difference was found between the GaAlAs laser treatment group and the placebo group [15]. There is no information in the literature on the use of the Nd:YAG laser in TN, and, in our study, LLLT with the Nd:YAG laser was also effective in reducing pain; however, a 31-point reduction in pain intensity occurred in the GRR laser group, whereas a 16-point reduction occurred in the Nd:YAG laser group.

The duration and number of sessions of LLLT for TN also vary across studies. In our study, we applied GRR laser treatment for a total of 25 sessions in 2 stages according to the manufacturer’s instructions and Nd:YAG laser treatment for a total of 12 sessions according to reference studies [10]. For the treatment of pain in TN, Ebrahimi et al. applied a total of 9 sessions 3 times per week and obtained effective results [14]. As in our study, Al-Azab et al. applied it 3 days per week for a total of 24 sessions, with each session lasting 20 min [15]. In other studies, a total of 9–12 sessions were generally used [10]. Further research is needed to optimize LLLT parameters, including laser type, wavelength, intensity, and treatment duration, to adapt treatment protocols to individual patients’ requirements.

Pain in TN is multidimensional and chronic, so evaluating pain reduction following treatment is more complicated. The VAS is commonly used in studies to assess pain intensity. The VAS is a pain scale with proven reliability that has been used for many years to measure pain in various conditions [25,26]. However, the VAS can measure only one aspect of pain, which is the intensity of pain at the time that the patient completes the questionnaire. Therefore, many scales have been developed to evaluate pain in the TN and facial region. The Barrow Neurological Institute Pain Intensity Scale (BNI-PS), the McGill Pain Questionnaire (MPQ), the Penn Facial Pain Scale (PFPS), and the Brief Pain Inventory (BPI-facial) are some of the scales used in studies [27]. Considering that the assessment of the patient’s immediate pain would not be sufficient to determine the efficacy of the treatment and to evaluate the improvement in the patient’s quality of life, we used the BPI-facial scale in our study. The BPI-facial is a scale developed according to the recommendations of the multi-institutional Initiative on Methods, Measurement, and Pain Assessment in Clinical Trials (IMMPACT), which is specifically based on facial pain [18,28]. The reliability and validity of this scale have been analyzed in many studies [29,30,31,32]. In his study, Burchiel evaluated the treatment outcomes of patients diagnosed with TN and tested the psychometric properties of the BPI-facial [33]. In our study, we assessed the PF in addition to the BPI-facial scale because the number of pain attacks is as important as pain intensity in TNs, and no studies in the literature have evaluated all these parameters.

In the literature, there are no studies on the new-generation laser device (GRR laser) in TN and, according to the clinical results we obtained, it was very effective in reducing symptoms. This laser system provides promising evidence in patients with TN when used as an adjunctive treatment. There was no change in all four variables in the placebo group. This suggests that LLLT is also effective in TN. While effective results were obtained after the treatments, long-term studies are necessary to assess the long-term effectiveness of LLLT in managing TN, including potential for pain relapse and the need for maintenance therapy.

## 5. Conclusions

In conclusion, a reduction in all the parameters was observed following LLLT. According to the reduction scores, the improvement in PF, PI, and IFA values was greatest in the GRR laser group. The GRR laser treatment was more effective than the Nd:YAG laser in reducing pain and improving quality of life in patients with TN. The LLLT can be used as an alternative treatment for TN to reduce the dose of medication and side effects of medical treatment.

## Figures and Tables

**Table 1:** Comparison of mean pre- and post-treatment variables in GRR laser group.

Paired Samples Statistics ^a^							
		Mean	N	Std. Deviation	Std. Error Mean	*t*	*p*
Pair 1	PF1	6.13	15	3.603	0.930	7.075	0.001
	PF2	0.80	15	1.082	0.279		
Pair 2	PI1	38.87	15	1.060	0.274	47.628	0.001
	PI2	7.73	15	2.404	0.621		
Pair 3	IGA1	39.13	15	6.300	1.627	7.685	0.001
	IGA2	33.13	15	5.617	1.450		
Pair 4	IFA1	57.40	15	6.162	1.591	22.877	0.001
	IFA2	17.40	15	6.390	1.650		
^a^ Group = GRR Laser							

**Table 2:** Comparison of mean pre- and post-treatment variables in Nd:YAG laser group.

		Mean	N	Std. Deviation	Std. Error Mean	*t*	*p*
Pair 1	PF1	6.80	15	4.127	1.065	5.512	0.001
	PF2	2.87	15	1.885	0.487		
Pair 2	PI1	39.00	15	0.926	0.239	11.985	0.001
	PI2	23.53	15	4.882	1.261		
Pair 3	IGA1	38.07	15	4.949	1.278	8.783	0.001
	IGA2	32.93	15	4.317	1.115		
Pair 4	IFA1	54.40	15	5.207	1.344	10.947	0.001
	IFA2	31.87	15	4.486	1.158		
a. Group = Nd:YAG Laser							

**Table 3:** Comparison of mean pre- and post-treatment variables in placebo group.

		Mean	N	Std. Deviation	Std. Error Mean	*t*	*p*
Pair 1	PF1	5.93	15	3.693	0.954	2.400	0.015
	PF2	4.40	15	1.882	0.486		
Pair 2	PI1	38.67	15	1.113	0.287	4.750	0.001
	PI2	36.27	15	2.017	0.521		
Pair 3	IGA1	39.33	15	8.415	2.173	2.331	0.018
	IGA2	37.67	15	7.118	1.838		
Pair 4	IFA1	51.93	15	6.573	1.697	2.988	0.005
	IFA2	49.13	15	6.823	1.762		
a. Group = Placebo							

## Data Availability

The original contributions presented in the study are included in the article, further inquiries can be directed to the corresponding author.
